# Körper und politische (An‑)Ordnungen. Zur Bedeutung von Körpern in der modernen westlichen Politischen Theorie

**DOI:** 10.1007/s11615-021-00357-4

**Published:** 2021-10-29

**Authors:** Gundula Ludwig

**Affiliations:** Center Interdisziplinäre Geschlechterforschung Innsbruck (CGI), Universität Innsbruck, Karl-Schönherr-Straße 3, 6020 Innsbruck, Österreich

**Keywords:** Politische Theorie, Ideengeschichte, Ausschlüsse, Legitimation, Politische Ordnung, Subjekt, Political theory, History of ideas, Exclusions, Legitimization, Political order, Political subjectivity

## Abstract

Die moderne westliche Politische Theorie befasst sich kaum mit Köpern; diese werden zumeist privatisiert und als natürlich bzw. vorpolitisch gesetzt. Der Text zeigt, dass Körper in der modernen Politischen Theorie allerdings nicht schlicht abwesend sind, sondern eine gewichtige politische Rolle einnehmen, denn Körper legitimieren politische Anordnungen in subtiler Weise. Durch eine Auseinandersetzung mit zentralen Denkfiguren bei Thomas Hobbes, John Locke, Jean-Jacques Rousseau, Immanuel Kant, Hannah Arendt, John Rawls und Jürgen Habermas werden drei Weisen sichtbar gemacht, wie Körper die moderne westliche Politische Theorie prägen: erstens werden Körper zur Legitimation der politischen Ordnung herangezogen, zweitens dienen sie der Bestimmung des politischen Subjektstatus und drittens wird über Körper Politik definiert. Der Text verdeutlicht, wie eine körpertheoretische Perspektive, die Körper nicht als präpolitisch, sondern als politisches Konstrukt begreift, den machtanalytischen Radius der Politischen Theorie zu erweitern in der Lage ist.

## Abstract (en)

At first glance, it can be stated that bodies do not play an important role in modern Western political theory. They are mostly privatized and set as natural or prepolitical. This article argues, however, that bodies are not absent in modern political theory but that they play a crucial political role. They legitimize political orders in a subtle way. Through an examination of central arguments in the work of Thomas Hobbes, John Locke, Jean-Jacques Rousseau, Immanuel Kant, Hannah Arendt, John Rawls, and Jürgen Habermas, three ways are identified in which bodies shape modern Western political theory. First, bodies are used to legitimize the political order; second, they serve to determine political subjectivity; and third, body politics define politics. The text aims to highlight how a body-theoretical perspective that does not define the body as prepolitical but rather as a political construct is able to expand the scope of political theory.

## Einleitung

Wenn einschlägige Einführungswerke als Indikatoren für die Antwort auf die Frage nach dem Gegenstandsbereich einer Disziplin herangezogen werden, dann zeigt sich, dass sich die Politische Theorie zwar um die Frage dreht, wie menschliches Zusammenleben gut, gerecht oder vernünftig gestaltet werden kann, dass aber die Subjekte, die dabei die Bühne der Politischen Theorie betreten, zumeist als körperlose Wesen imaginiert werden.^1^ „Die moderne politische Theorie hat den Körper weitgehend privatisiert“ (Kerner 2013, S. 113), bemerkt Ina Kerner treffend. Paula Diehl weitet diesen Befund auf die gesamte Politikwissenschaft aus und hält für diese fest, dass der Körper in keiner Weise zu deren „gängigen Gegenstand“ zählt (Diehl 2014, S. 116). Allerdings wäre es, so die Ausgangsüberlegung dieses Textes, verkürzt, zu konstatieren, dass Körper in der modernen Politischen Theorie schlicht abwesend sind. Obgleich sich die Politische Theorie nicht explizit mit Körpern befasst, nehmen diese eine zentrale Rolle ein – in zweierlei Hinsicht und jeweils auf unterschiedliche Art und Weise: als politischer Körper in der Imagination und Konstruktion der politischen Ordnung und als leiblicher Körper, über den Ein- und Ausschlüsse sowohl in der Definition des politischen Subjekts als auch von der Politik legitimiert werden. Diesen Bezügen ist gemein, dass Körper als *präpolitische* Setzungen fungieren, die *politische* Anordnungen legitimieren.

Das Augenmerk darauf gelenkt zu haben, dass auch das vermeintlich Präpolitische zutiefst politisch ist, ist zentrales Verdienst feministischer, postkolonialer, ability-zentrismuskritischer und queerer Politischer Theorien. Diese haben durch das Sichtbarmachen von Macht- und Herrschaftsverhältnissen im vermeintlich Privaten, wie etwa in sozialen Beziehungen, im Alltag oder in der Sexualität, den analytischen Radius Politischer Theorie erweitert (u. a. Arneil und Hirschmann 2016a; Eze 1997a; Lyndon und Pateman 1991). Zudem haben sie Instrumentarien erarbeitet, um zu erfassen, wie das vermeintlich Präpolitische mit Politik und der politischen Ordnung konstitutiv verwoben ist – beispielsweise im Verhältnis von Privatheit und Öffentlichkeit oder in der Bedeutung der Familie für die Architektur des modernen westlichen Staates (Fraser 1985; Sauer 1997).

Die feministischen, queeren, ability-zentrismuskritischen und postkolonialen Erweiterungen der Politischen Theorie lassen sich entlang dreier Analysedimensionen bündeln: Erstens legen sie den Fokus darauf, wie politische Ordnung durch Konstruktionen von Geschlecht, Sexualität, „Behinderung“ und *race* geprägt sind, die jedoch im Kanon der Politischen Theorie und Ideengeschichte selbst naturalisiert werden und folglich als vermeintlich präpolitische Konstituens Theorien über den Gesellschaftsvertrag, den Staat oder Demokratie prägen. So haben beispielsweise Carole Pateman (1988) und Charles Mills (1997) in ihrer feministischen bzw. postkolonialen Re-Lektüre der Vertragstheorien den Nachweis erbracht, dass der Gesellschaftsvertrag auf einem Geschlechtervertrag und einem *racial contract* beruht, was zugleich durch die Naturalisierung von Geschlecht und *race* entpolitisiert wird.

Zweitens haben diese kritischen Interventionen sichtbar gemacht, dass sich die Konzeption des politischen Subjekts in der modernen westlichen Denktradition heteronormativer, andro-, ability- und eurozentrischer Konstruktionen bedient. Hier wurde nicht nur die Kompliz*innenschaft des Kanons der Politischen Theorie mit real-politischen Ausschlüssen von nicht-männlichen, nicht-weißen, nicht-heteronormativen und „behinderten“ Menschen aus Öffentlichkeit und Politik freigelegt (u. a. Kreisky 1995; Dhawan 2014; Young 1990). Darüber wurde aufgezeigt, wie die Konstruktion des politischen Subjekts als „rationales“ und „autonomes“ Wesen die Definition des politischen Subjekts ganz grundsätzlich verengt (u. a. Butler 2005; Quijano 2000). Bedürfnisse, Abhängigkeiten und Emotionen werden in einer derartigen Konstruktion keinerlei Bedeutung zugemessen und wurden daher als präpolitisch konfiguriert und historisch auf jene Menschen ausgelagert, die zu den „Anderen“ der politischen Subjekte gemacht wurden: *People of Color, *Schwarze Menschen, Indigene Menschen, Frauen, Lesben, Schwule, Queers und Menschen mit Beeinträchtigungen.

Drittens haben diese Ansätze dargelegt, dass die Konzeption von Politik auf ability-, andro-eurozentrischen und heteronormativen Prämissen beruht. Politik wird in der Denktradition der westlichen modernen Politischen Theorie als vernunftbasiertes und -zentriertes Handeln in der Öffentlichkeit bestimmt. Auf diese Weise wird jedoch die Konzeption von Politik verengt: Zum einen, da „Vernunft“, wie u. a. Seyla Benhabib (1992b), Nikita Dhawan (2014) und Iris Marion Young (1990) argumentierten, in einer herrschaftsförmig organisierten Gesellschaft Ausdruck und Sedimentierung von Erfahrungen hegemonialer Subjektpositionen ist und politisches Handeln von marginalisierten Menschen daher oftmals als „irrational“ und „partikular“ abgewertet wird. Zum anderen werden all jene gesellschaftlichen Bereiche – wie etwa Sorge- und Pflegetätigkeiten –, die nicht mittels Vernunft organisiert werden können, aus einem derartigen Verständnis von Politik ausgeschlossen und in die vermeintlich „unpolitische“ Sphäre der Privatheit verbannt (Fraser 1996; Nussbaum 2010).

Mit vielfältigen Reflexionen entlang dieser drei Dimensionen haben queere, feministische, ability-zentrismuskritische und postkoloniale Theorien gezeigt, wie vermeintlich präpolitische Geschlechter-, dis/ability-, sexuelle und rassifizierende Verhältnisse grundlegend in die Ausgestaltung der politischen Ordnung, des politischen Subjekts und der Politik eingeschrieben sind. Diese Interventionen führten zu einer Ausweitung des analytischen Radius der Politischen Theorie, indem sichtbar gemacht wurde, wie Macht- und Herrschaftsverhältnisse im vermeintlich Präpolitischen überaus subtil, dafür umso wirkmächtiger ihre Wirkweise entfalten können, und wie Politik verengt wird, wenn manche Tätigkeiten und Praktiken ausgeschlossen werden, indem sie der Kanon der Politischen Theorie als unbedeutend, da „unpolitisch“ definiert.

Diese queere, feministische, postkoloniale, ability-zentrismuskritische Epistemologie liegt auch dem vorliegenden Text zugrunde. Mit dieser ausgestattet möchte ich mich der Bedeutung von Körpern in der modernen westlichen Politischen Theorie zuwenden. Ich übernehme im Folgenden daher die eben skizzierten drei Analysedimensionen und beziehe sie auf das Anliegen, die Kritik an den Begrenzungen in der westlichen Politischen Theorie zu vertiefen, indem ich das Augenmerk auf die spezifische Rolle von Körpern dabei richte. Das Ziel des Textes besteht darin, sichtbar zu machen, dass Körper eine wichtige Bedeutung für die Legitimation von Begrenzungen und Ausschlüssen einnehmen – bezogen auf die Konzeption politischer Ordnung, der politischen Subjekte und von Politik – nicht obwohl, sondern *gerade weil *sie im Kanon der Politischen Theorie als *„naturgegeben“ und präpolitisch* gelten.

Die nachfolgende Darstellung ist keine umfassende Systematik über Körper in der modernen westlichen Politischen Theorie. Vielmehr möchte der Text die Breite der Bedeutung zeigen, die Körper im politischen Denken einnehmen, indem zentrale Theorien darauf hin befragt werden, wie die Konstruktion der politischen Ordnung, des politischen Subjekts und von Politik mit Konstruktionen von Körpern verwoben wird. Auf diese Weise soll sichtbar gemacht werden, dass Körper in vielfältigen Formen eine gewichtige Rolle in der Politischen Theorie einnehmen – in den klassischen Vertragstheorien von Hobbes, Locke und Rousseau ebenso wie in Kants Theorie des politischen Subjekts sowie in durchaus diametralen Konzeptionen von Politik, wie sie sich in zeitgenössischen Ansätzen von Arendt, Habermas und Rawls finden lassen. Die Auswahl der Autor*innen ist derart gewählt, dass wichtige Vertreter*innen des Kanons der Politischen Theorie aus heterogenen Theorietraditionen und mit unterschiedlichen Schwerpunkten herangezogen werden, um zu zeigen, dass Körper in ganz unterschiedlichen Denktraditionen – vertragstheoretischen, republikanischen, deliberativen und liberalen – eine zentrale *politische Rolle* spielen. Angesichts dieser gewichtigen Bedeutung von Körpern für politische (An‑)Ordnungen plädiert der Text dafür, dass es in der Politischen Theorie höchst an der Zeit ist, kritische Auseinandersetzungen mit Körpern von ihren Rändern in ihr Zentrum vordringen zu lassen.

## Körper und die politische Ordnung

In der modernen westlichen Politischen Theorie gilt nicht die ungezügelte vielstimmige Menge als Garant der politischen Ordnung, sondern eine durch Vernunft entstandene Einheit. Diese Vorstellung, verweist, wie Martin Saar (2013) rekonstruierte, darauf, dass sich die Hobbes’sche Tradition gegen die Spinoza’sche Tradition durchsetzte und Hobbes’ Unterscheidung zwischen Volk und Menge prägend für das moderne westliche Denken über die politische Ordnung wurde: „Das *Volk*“ gilt Hobbes als „Einheit mit *einem* Willen“ (Hobbes 1918, S. 206), das gerade weil es vereinheitlicht ist, zu Verträgen und rechtlichem Handeln fähig wird. Demgegenüber ist die Menge der Inbegriff von Chaos, Unregierbarkeit und Bedrohung der politischen Ordnung, denn die Menge sind „nicht eine Körperschaft, sondern viele Menschen, von denen jeder seinen eigenen Willen und sein eigenes Urteil über alles hat, was vorgebracht wird“ (Hobbes 1918, S. 136). Nicht die Menge, die „multitudo“, ist ordnungsbegründend; diese wird zum Synonym für Chaos, Unregierbarkeit und Bedrohung der Ordnung und muss im „Volkskörper“ gezähmt werden (Lorey 2012, S. 28; Saar 2013, S. 355).

In dieser Begründung politischer Ordnung als Einheit findet sich bei Hobbes ein doppelter Rückgriff auf den Körper – einmal auf den leiblichen, einmal auf den politischen Körper. Hobbes leitet die Notwendigkeit eines vereinten Souveräns aus seiner Konzeption des Naturzustands als Zustand eines „Krieg[es] eines jeden gegen jeden“ (Hobbes 1994, S. 96) ab. Dass der Naturzustand einem Kriegszustand gleicht, begründet Hobbes mit naturgegebenen Trieben der Menschen. Da dem Verlangen „nach einem angenehmen Leben und nach Sinnenfreuden“ (Hobbes 1994, S. 76) zerstörerische Kraft zukomme, seien die Einsetzung eines Souveräns und die Unterwerfung unter diesen unabdingbar (Hobbes 1994, S. 131). Es ist also die *körperliche* Verletzbarkeit und die *körperliche* Gefährdung, die es nach Hobbes erforderlich machen, den Naturzustand zu verlassen und in eine politische Ordnung einzutreten. Der „Trieb“ der Selbsterhaltung tritt bei Hobbes zugleich *für* die Natur – das Leben – als auch *gegen* die Natur – den Tod – ein. Aufgrund dieses Doppelcharakters kann Hobbes aus der körperlichen Natur sowohl die Unordnung im Naturzustand als auch den Willen für ihre Überwindung ableiten. Auf „Antrieb der Natur“ (Hobbes 1994, S. 159) würden Menschen sich verpflichten, in den vertraglichen Rechtszustand einzutreten, der sie zugleich aus ihrer Natur befreien würde. Die Referenz auf den leiblichen Körper mit seinen „naturgegebenen Trieben“ und der „natürlichen“ Vernunft dient Hobbes als Begründung sowohl für den kriegerischen Naturzustand als für die Notwendigkeit, diesen zu überwinden.

Die zweite körpertheoretische Argumentationsfigur in Hobbes’ politischem Denken zielt darauf ab, den souveränen Staat als einheitsstiftende Instanz zu legitimieren und politische Ordnung mit Einheit gleichzusetzen. An dieser Stelle wechselt Hobbes von der Referenz auf den leiblichen zum „politische[n] Körper“ (Hobbes 1994, S. 5): Während der Körper im Naturzustand gerade als leiblicher gefährdet ist, verspricht der Souverän als politischer Körper Schutz und Sicherheit. Die Fähigkeit des politischen Körpers, das „Volk“ zu schützen, entsteht nach Hobbes gerade dadurch, dass dieser ein aus allen vereinter Körper ist (Hobbes 1994, S. 134). Diese Einheit entwirft Hobbes über eine Analogie zum menschlichen Körper. Der *Leviathan* als künstliche Nachahmung der Natur (Hobbes 1994, S. 134) wird durch die metaphorische Koppelung an den natürlichen Körper legitimiert.

Die *Souveränität* stellt darin eine künstliche *Seele* dar, die dem ganzen Körper Leben und Bewegung gibt, die *Beamten *und anderen* Bediensteten* der Jurisdiktion und Exekutive künstliche *Gelenke, Belohnung *und *Strafe*, die mit dem Sitz der Souveränität verknüpft sind und durch die jedes Gelenk und Glied zur Verrichtung seines Dienstes veranlaßt wird, sind die *Nerven*, die in dem natürlichen Körper die gleiche Aufgabe erfüllen. […] *Eintracht *ist* Gesundheit, Aufruhr, Krankheit *und* Bürgerkrieg Tod.* (Hobbes 1994, S. 5)

Hobbes’ Staatsbegründung enthält folglich das Versprechen, die Verletzbarkeit der leiblichen Körper durch die Unterwerfung des Souveräns zu überkommen, und darüber hinaus wird die Sicherheit, die der politische Körper des Souveräns den Unterworfenen verspricht, durch eine Anleihe an eine Körperanalogie untermauert: So wie der „natürliche Körper“ als Gleichgewicht und Harmonie funktioniert, so auch der *Leviathan*. Der „natürliche“ und präpolitische Körper ist für Hobbes also gesichertes wie sicherndes Wissen, das er zum Fundament der politischen Ordnung macht.

Auch bei John Locke findet sich eine ähnliche doppelte Bezugnahme auf den Körper zur Legitimation der gesellschaftlichen Ordnung: Obgleich Locke den Naturzustand nicht wie Hobbes als Kriegszustand konzeptualisiert, schreibt auch er dem leiblichen Körper eine wichtige Bedeutung im Naturzustand zu: Bei Locke ist der Selbsterhaltungstrieb des Menschen im Naturzustand grundlegend (Locke 1977, S. 136). Allerdings stellt Locke nicht die unmittelbare Gefährdung der leiblichen Körper ins Zentrum, sondern die Gefährdung des Eigentums und der natürlichen Freiheit – und dieses leitet er aus dem Körper ab (Locke 1977, S. 216). Dieses grundlegende Eigentumsverhältnis über den Körper dient Locke als Rechtfertigung des allgemeinen Eigentums:

Obwohl die Erde und alle niederen Lebewesen allen Menschen gemeinsam gehören, so hat doch jeder Mensch ein *Eigentum* an seiner eigenen *Person*. Auf diese hat niemand ein Recht als nur er allein. Die *Arbeit* seines Körpers und das Werk seiner Hände sind, so können wir sagen, im eigentlichen Sinne sein Eigentum. Was immer er also dem Zustand entrückt, den die Natur vorgesehen und in dem sie es belassen hat, hat er mit seiner *Arbeit *gemischt und ihm etwas eigenes hinzugefügt. Er hat es somit zu seinem *Eigentum* gemacht. (Locke 1977, S. 216)

Das Selbst-Eigentum am Körper begründet Locke zufolge die natürliche Freiheit im Naturzustand, aber zugleich liegt im Fehlen einer übergeordneten Instanz die Gefahr für diese, sodass es im Naturzustand keine dauernde Sicherung der Freiheit der Einzelnen geben kann (1977, S. 204). Während es bei Hobbes ganz unmittelbar die leiblichen Körper sind, deren Gefährdung den Ausgang aus dem Naturzustand erfordern, nehmen die Körper bei Locke eine vermittelte Bedeutung über die Freiheit und das Eigentum ein, die diese begründen. Um das Selbst-Eigentum am Körper, ihre Freiheit und ihr Eigentum an Gütern zu sichern, unterwerfen sich nach Locke die Menschen dem Gesellschaftsvertrag, der das „behagliche, sichere und friedliche Miteinanderleben“ sichere (Locke 1977, S. 260). Auch Locke begründet daher aus der Gefährdung des aus den Körpern abgeleiteten Eigentums die Notwendigkeit einer politischen Macht, deren Zweck die Sicherung des Eigentums sein soll (Locke 1977, S. 278).

Die politische Ordnung, die durch den Gesellschaftsvertrag hergestellt werden soll, konzipiert Locke ebenso als Einheit – und auch ihm dient ein Rekurs auf den Körper dabei als Legitimationsquelle. In seinen *Zwei Abhandlungen über die Regierung* konturiert er die politische Gemeinschaft als einen vereinheitlichen Körper: „Wenn eine Anzahl von Menschen darin *eingewilligt hat, eine einzige Gemeinschaft *oder eine Regierung zu bilden, so haben sie sich ihr damit gleichzeitig einverleibt, und sie bilden *einen einzigen politischen Körper*“ (Locke 1977, S. 260). Die Einheit des politischen Körpers müsse wiederum durch das Mehrheitsprinzip sichergestellt werden, dem die *Bürger* mit dem Gesellschaftsvertrag zustimmen.^2^

Denn da eine Gemeinschaft allein durch die Zustimmung ihrer einzelnen Individuen zu handeln vermag und sich ein einziger Körper auch nur in einer einzigen Richtung bewegen kann, so muß sich notwendigerweise der Körper dahin bewegen, wohin die stärkere Kraft ihn treibt. Und das eben ist die *Übereinstimmung der Mehrheit*. Andernfalls wäre es unmöglich, daß die Gemeinschaft als ein Körper, als eine *einzige Gemeinschaft* handeln und fortbestehen kann, wie es doch durch die Zustimmung aller Individuen, die sich in ihr vereinigt haben, beschlossen worden war. (Locke 1977, S. 260)

Das Mehrheitsprinzip ist für Locke alternativlos, denn ohne dieses würde es den politischen Körper zerreißen (Locke 1977, S. 260). Auch Locke begründet daher die Notwendigkeit eines politischen Körpers als Einheit aus den – über das Eigentum vermittelten – leiblichen Körper und deren Gefährdung im Naturzustand.

Die Vorstellung der politischen Ordnung als Einheit findet sich auch bei Jean-Jacques Rousseaus wieder – ebenso wie dafür eingesetzte Körpermetaphern. Aus republikanischer Tradition begründet Rousseau jedoch die Notwendigkeit des Gesellschaftsvertrags nicht wie Hobbes und Locke mit der Sicherung des leiblichen Körpers respektive des Eigentums an und aus diesem. Dem leiblichen Körper und dessen potenzielle Gefährdung kommen bei Rousseau keine argumentative Bedeutung in der Begründung der Volkssouveränität zu. Der Körper betritt bei Rousseau erst die argumentative Bühne, wenn vom politischen Körper der Volkssouveränität die Rede ist. Denn diese legitimiert auch Rousseau als Einheit, indem er den politischen Körper mit dem natürlichen Körper gleichsetzt: Wie die einzelnen Glieder eines Körpers haben sich auch die Subjekte unter den Gesellschaftsvertrag als eine Einheit hervorbringende Institution unterzuordnen.

*Gemeinsam stellen wir alle, jeder von uns seine Person und seine ganze Kraft unter die oberste Richtschnur des Gemeinwillens; und wir nehmen, als Körper, jedes Glied als untrennbaren Teil des Ganzen auf. *Dieser Akt des Zusammenschlusses schafft augenblicklich anstelle der Einzelpersonen jedes Vertragspartners eine sittliche Gesamtkörperschaft, die aus ebenso vielen Gliedern besteht, wie die Versammlung Stimmen hat, und die durch ebendiesen Akt ihre Einheit, ihr gemeinschaftliches Ich, ihr Leben und ihren Willen erhält. (Rousseau 2003, S. 18)

Durch den Gesellschaftsvertrag würden sich die Menschen als „vereint als eine einzige Körperschaft betrachten“ (Rousseau 2003, S. 112), die „nur einen einzigen Willen [haben], der sich auf die gemeinsame Erhaltung und auf das allgemeine Wohlergehen bezieht. In diesem Zustand sind alle Triebkräfte des Staates gesund und einfach, seine Grundsätze sind klar und einleuchtend“ (Rousseau 2003, S. 115).

Der Souverän als aus allen Einzelnen hervorgegangene Einheit habe daher „auch kein dem ihren [der Einzelnen] widersprechendes Interesse“ (Rousseau 2003, S. 20). Für den Fall wiederum, dass die Interessen der Einzelnen dem gemeinsamen Interesse des Souveräns entgegenstreben, ermächtige der Gesellschaftsvertrag die Anderen dazu, „daß, wer immer sich weigert, dem Gemeinwillen zu folgen, von der gesamten Körperschaft dazu gezwungen wird, was nichts anderes heißt, als dass man ihn zwingt, frei zu sein“ (Rousseau 2003, S. 21). Die Einheit des Souveräns, für die Rousseau argumentiert, ist freilich nicht wie bei Hobbes der Staat, sondern das „Volk“ (Rousseau 2003, S. 40). Dennoch rekurriert auch Rousseau auf den leiblichen Körper als Ideal für den politischen Körper, um das Ideal der Einheit zu rechtfertigen.

Der Einsatz einer kritischen Befragung von Körpern in der Politischen Theorie ist nun folgender: Wenn wir, wie von queeren, feministischen, ability-zentrismus-, rassismus- und eurozentrismuskritischen Theoretiker*innen unternommen, den Vertragstheoretikern nicht in ihrer Setzung folgen, dass es einen „natürlichen Körper“ gibt, der jenseits von Politik existiert, kann die Frage gestellt werden, welcher „Körper“ den Vertragstheoretikern eigentlich als Referenz für den „leiblichen“ sowie „politischen“ Körper dient. Aus dieser Perspektive lässt sich die Konzeption des leiblichen Körpers, der im Hobbes’schen Naturzustand direkt und im Locke’schen indirekt, als gefährdet gilt, bereits als machtvolle Konstruktion entlarven. Hobbes leitet aus der Vulnerabilität der „leiblichen Körper“ die Notwendigkeit der Unterwerfung unter den Souverän ab und begründet damit eine Argumentationslinie, die bis in die Gegenwart das Verständnis von politischer Ordnung prägt: Ziel des Souveräns müsse sein, Sicherheit und Schutz der Körper zu gewährleisten. Damit wird die Notwendigkeit der Unterwerfung aus der Vulnerabilität der leiblichen Körper legitimiert, der als präpolitisch verhandelt wird. Wie Judith Butler (2005) jedoch argumentiert, impliziert dies bereits eine *überaus politische Setzung über „den Körper*“: Nach Butler ist Vulnerabilität nicht ein naturgegebener Makel oder Mangel der leiblichen, präpolitischen Körper, der überwunden werden müsse, sondern vielmehr eine Bedingung von Leben (Butler 2005, S. 46). Diese fundamentale Vulnerabilität von Menschen leitet Butler nicht aus der Sterblichkeit der Körper ab, sondern aus deren Sozialität und verortet die Vulnerabilität von Körpern nicht in einem vermeintlich vorpolitischen Raum, sondern bereits im politischen. Wie Butler hervorhebt, gilt Vulnerabilität seit Hobbes in der Politischen Theorie als naturgegebene Bedrohung, die durch einen Souverän überwunden werden müsse. Die Vulnerabilität, so lässt sich mit Butler gegen Hobbes argumentieren, resultiert aber gerade nicht aus den vermeintlich präpolitischen leiblichen Körpern, sondern aus *politischen Bedingungen*, die dazu führen, dass (manche) Körper mittels Grenzziehungen über Geschlecht, Heteronormativität, *race*, nationalstaatliche Zugehörigkeit, Klasse und Ability als weniger schützenswert gelten als andere. Die Konstruktion von Vulnerabilität der leiblichen Körper als *naturgegebene Bedrohung* legt jedoch seit Hobbes den Grundstein für eine Konzeption von politischer Ordnung als Ordnung der Souveränität, die den Schutz mancher unter der Bedingung der Unterwerfung unter den Souverän verspricht. Indem Hobbes die Vulnerabilität als naturgegebene Gefahr aus den „natürlichen“ Körpern ableitet, verschleiert er, dass diese grundlegende Konzeption „leiblicher Körper“ bereits selbst zutiefst politisch ist.

Auch Locke arbeitet mit einer zutiefst politischen Prämisse, wenn er über die „natürlichen“ Körper schreibt: Denn die Annahme, dass Körper Eigentum der Subjekte seien, lässt sich keineswegs aus den leiblichen Körpern ableiten, sondern ist eine politische Annahme. Mit Butler kann Locke hier entgegnet werden, dass Körper ein „Modus einer Beziehung“ zu anderen Körpern und Subjekten und als solches ein „Modus der Enteignung“ sind (Butler 2005, S. 41). Anders als bei Locke ist Butlers Perspektive auf den Körper jedoch keine naturalistische Ontologie, sondern eine soziale Ontologie, eine – diametral zur Bedeutung, die die Ontologie der Körper in den Vertragstheorien von Hobbes und Locke einnimmt – in politischen Verhältnissen gewordene „Ontologie“. Diese soziale Ontologie des Körpers verweist auf „Gefährdung, Schutzlosigkeit, Verletzlichkeit, wechselseitige Abhängigkeit, Exponiertsein“ (Butler 2010, S. 10). Gerade diese soziale Ontologie von Körpern ignoriert Locke jedoch und leitet aus der vermeintlich naturgegebenen Ontologie des leiblichen Körpers als Eigentum des Subjekts ebenso die Notwendigkeit des Gesellschaftsvertrags, der einheitlichen Ordnung und der Unterwerfung unter diese ab.

Auch auf der Ebene des politischen Körpers zeigt sich, dass die Vertragstheoretiker dabei nur vermeintlich auf einen „naturgegebenen“, präpolitischen Körper rekurrieren. Insbesondere bei Hobbes und bei Rousseau lässt sich nachzeichnen, dass die durch Analogie mit dem „leiblichen“ Körper hergestellte „Einheit“ des politischen Körpers immer fragil ist (Hobbes 1994, S. 136; Rousseau 2003, S. 49). Sie wird nicht ein für alle Mal hervorgebracht, sondern muss immer wieder her- und sichergestellt werden. Es ist, so lässt sich argumentieren, eine ganz spezifische Konstruktion des „leiblichen Körpers“, die in den Vertragstheorien als *argumentative Strategie der Versicherung und Sicherheit* herangezogen wird, um diese Einheit des politischen Körpers zu stabilisieren. Wie feministische, queere, ability-zentrismus-, rassismus- und eurozentrismuskritische Theoretiker*innen argumentiert haben, ist die für die Legitimation politischer Ordnung als Einheit so zentrale Figur des Körpers als Einheit nicht etwas, was sich aus den „natürlichen Körpern“ ableiten lässt, sondern eine zutiefst politische Konstruktion. Denn der „leibliche“ Körper ist keine abgegrenzte, substanzielle Einheit, sondern vielmehr ein interkorporeales Gefüge, das erst in Beziehungen zu anderen Körpern, Subjekten, Institutionen zu einem je spezifischen Körper *wird* (Fritsch 2010, S. 7). Der Körper ist ein in sozialen Bezügen und in historisch-spezifischen Macht-Wissens-Verbindungen *Werdendes*. „The body is not a contained individual but an assemblage of organs, processes, pleasures, passions, activities, and behaviors“ (Fritsch 2010, S. 7), „a set of operational linkages and connections with other things, other bodies“ (Grosz 1994, S. 120). Die Vorstellung des Körpers als Einheit ist folglich ein *Phantasma*, das andro-, euro-, ability-zentrische und heteronormative Setzungen verlangt (u. a. Braidotti 1997; Butler 1995; Grosz 1994; Shildrick 1996): Das Phantasma des Körpers als Einheit setzt voraus, dass alles, was diese Konstruktion bedroht, zum „Abjekt“ (Kristeva 1982, S. 71) gemacht werden muss. Geschlecht, *race*, Sexualität und Behinderung fungieren hier als diskursive Konstrukte, die Grenzziehungen zwischen jenen Körpern vollziehen, die als Einheit konzipiert werden, und Körpern, die als entgrenzt, fluide, unkontrollierbar und als Bedrohung der Einheit gelten.

Vor diesem Hintergrund dechiffriert Moira Gatens (1997) den „einheitlichen Körper“, wie er in der modernen westlichen Politischen Theorie als Referenz für die politische Ordnung bedient wird, als maskulinen, weißen Körper, als „fantasy of unity“ (Gatens 1997, S. 87). Diese phantasmatische Konstruktion des Körpers als Einheit bot den Vertragstheoretikern eine argumentative Referenz, um die Einheit des politischen Körpers als „natürliche“ politische Ordnung zu rechtfertigen. Die Gefahr, dass die Einheit des Souveräns als politischer Körper – als Staat oder Volkssouveränität – misslingen könnte, sollte durch den Rekurs auf den vermeintlich präpolitischen natürlichen Körper gebannt werden.

Die aus dem vermeintlich natürlichen Körper abgeleitete „Vorstellung einer einheitlichen geschlossen Macht, die weder Konflikte noch Differenzen zuläßt“ (Appelt 1999, S. 47), diente zugleich als politische Technik, um Ausschlüsse qua Vergeschlechtlichung, Rassifizierung, Klasse und Behinderung aus der phantasmatischen Einheit zu entpolitisieren. Carole Pateman zeigte dies in ihrer Kritik an der vergeschlechtlichen Logik des Gesellschaftsvertrags: „If women took part in the original contract the awesome figure of the mortal god Leviathan could not be created […]. No such unity would be possible if both sexes took part in the constitution of Leviathan – there could be no representative figure who could represent the ‚person‘, the bodily form of both sexes.“ (Pateman 1991, S. 68) Nicht nur ist also die Vorstellung des Körpers als Einheit ein Phantasma; die Referenz auf einen vermeintlich einheitlichen Körper dient(e) ebenso dazu, ein politisches Gemeinwesen trotz Ausschlüssen und Ungleichheiten als Einheit darzustellen. Die „Koppelung von Körperbildern mit Vorstellungen des Kollektiven“ (Harrasser 2009, S. 201) und damit das Anknüpfen an vermeintlich Natürliches ermöglicht(e) es, eine machtvolle Selbstimagination des Staates und der Souveränität hervorzubringen und eine vereinte politische Gemeinschaft zu instituieren und legitimieren.

## Körper und das politische Subjekt

Auch in der Bestimmung des politischen Subjektstatus kommt dem Körper eine emblematische Rolle zu – und zwar in zweierlei Hinsicht: Ein körpertheoretischer Blick in das Archiv der westlichen modernen Politischen Theorie macht sichtbar, dass die Herausbildung von politischer Subjektivität ein spezifisches Verhältnis zum Körper voraussetzt(e). Zugleich wurden – gleichsam als Kehrseite – manche Körper als Begründung für das Absprechen des politischen Subjektstatus herangezogen. Indem nachfolgend beide Dimensionen rekonstruiert werden, soll ein Spannungsfeld bezogen auf das Verhältnis von Körpern und politischem Subjekt aufgezeigt werden: Obwohl das politische Subjekt der westlichen Moderne als entkörpertes entworfen wird, nehmen machtvolle Imaginationen über den vergeschlechtlichten, rassifizierten, sexualisierten, „behinderten“ Körper in der Bestimmung, wer als politisches Subjekt zählt, ebenso wie in der Ausgestaltung des politischen Subjekts eine grundlegende Rolle ein.

Mit seiner spezifischen Verbindung von politischer Subjektivität, Körper und Eigentum legte Locke den Grundstein für das moderne Verständnis des *Bürgers*: Locke konstruiert den Körper als Gegenspieler der Vernunft, den diese bezwingen und kontrollieren muss, damit sich das Subjekt der Vernunft als *Bürger *qualifiziert. Der Körper wird somit zugleich zu einer bloßen Voraussetzung des politischen Subjektstatus wie zu jenem Terrain, auf dem die Vernunft des *Bürgers* über die Herrschaft über den Körper unter Beweis gestellt werden muss. Der Körper ist folglich *anwesend* als Voraussetzung für den politischen Subjektstatus, wird aber durch die bezwingende Vernunft *abwesend*. Diese Komposition des Verhältnisses von Vernunft und Körper zeitigte eine folgenreiche Konsequenz. Denn in Lockes Denkgebäude verfügen nicht alle Menschen über Eigentum über ihren Körper, weshalb er auch nicht allen die Fähigkeit, „sich selbst und andere zu regieren“ zuschreibt (Locke 1977, S. 237). Wie Charles W. Mills (1997, S. 67–68, 2008, S. 1382) rekonstruiert, beruht Lockes Argumentation auf einem *Racial Contract*: Schwarzen Menschen, Indigenen Menschen und *People of Color* spricht Locke gerade aufgrund ihrer Körper ab, politische Subjekte werden zu können (Locke 1977, S. 216). Die Vorstellung, dass Menschen über ein souveränes Eigentumsverhältnis über ihren Körper zu politischen Subjekten werden, ist eine zutiefst „racialized liberal norm“ (Mills 2008, S. 1382): Der „Racial Contract is explicitly predicated on a politics of the body which is related to the body politic through *restrictions* on which bodies are ‚politic‘. There are bodies impolitic whose owners are judged incapable of *forming* of fully *entering into* a body politic“ (Mills 1997, S. 53).^3^

Ebenso wenig gelten Locke Menschen, die er als außerhalb des „gewöhnlichen Verlauf[s] der Natur“ stehend definiert (Locke 1977, S. 236), als Eigentümer ihres Körpers. Auch „*Geisteskranke*“ und „*Idioten“* (Locke 1977, S. 236) schließt er aus dem Kreis der politischen Subjekte aus, wie auch Barbara Arneil problematisiert. „Because government requires rational consent, ‚lunaticks‘ and ‚ideots‘ are the opposite of ‚freemen‘ and therefore ruled, according to Locke, under a perpetual (rather than limited) form of ‚government‘ within the private/domestic (rather than public) sphere“ (Arneil 2009, S. 222).

Heteronormativ-patriarchale Setzungen in Lockes politischer Theorie führen schließlich dazu, dass Locke auch Frauen nicht als Eigentümerinnen ihrer Körper und folglich nicht als Bürgerinnen auffasste:

Women’s bodies were not susceptible to the sort of self-mastery required of a self-proprietor. Briefly put, women could not contain themselves for they were of their nature, it seemed, containers. Their bodies contained the male in the act of heterosexual sex and then they contained the future generation. Thus the state, as well as the community of men, must always retain a controlling interest in the female form. Women could not be endowed with exclusive property interests in their bodies when there were male interests in selfhood at stake. (Naffine 1998, S. 203)

Locke, der mit seiner Konzeption des politischen Subjekts die bis heute gültigen Parameter „des Selbstverständigungsdiskurses westlicher liberal-demokratischer Verfassungsstaaten“ bereitstellte (Brocker 2007, S. 271), legte folglich eine zutiefst *verkörperte* Definition des politischen Subjekts vor: Während weiße, besitzende, „nichtbehinderte“ Männer über ein Eigentumsverhältnis über ihre Körper zu politischen *Bürgern* werden können, wird dies all jenen, die zu *Anderen* gemacht wurden, gerade aufgrund der Unmöglichkeit eines Selbst-Eigentums am Körper abgesprochen. Barbara Arneil und Nancy Hirschmann folgern daher mit Blick auf Locke’s ableistische Setzungen: „The citizen […] is constituted in opposition to disabled dependents, outside the contract, who have needs that must be attended to as determined by the principles of charity and welfare“ (Arneil und Hirschmann 2016b, S. 7). Jene, die durch Vergeschlechtlichungs‑, Sexualisierungs‑, Behinderungs- und Rassifizierungsprozesse zu Anderen gemacht werden, *haben* daher nicht nur einen Körper, den sie zu kontrollieren angeblich nicht in der Lage seien, sondern *sind* zugleich stets auch Körper. Trotz dieser zentralen Bedeutung, die Körpern bei Locke für die Frage zukommt, wer *nicht *als Bürger*in gilt, können diese politischen Ausschlüsse entpolitisiert bleiben, da die „leiblichen Körper“ für Locke als präpolitisch gelten.

Eine weitere ideengeschichtlich folgenreiche Verzahnung von Körpern und politischem Subjektstatus zeigt sich bei Rousseau. Rousseau schreibt dem Gesellschaftsvertrag die Kraft zu, dass die Menschen durch diesen ihre körperliche Angewiesenheit ablegten.

Wer sich daran wagt, ein Volk zu errichten […], muß sich imstande fühlen, sozusagen die menschliche Natur zu ändern […], um sie zu stärken; an die Stelle eines physischen und unabhängigen Daseins, das wir alle von der Natur erhalten haben, ein Dasein als Teil und ein moralisches Dasein zu setzen. Mit einem Wort, es ist nötig, daß er dem Menschen die ihm eigene Kraft raubt, um ihm fremde zu geben, von denen er nur mit Hilfe anderer Gebrauch machen kann. (Rousseau 2003, S. 43–44)

Den Übergang vom Naturzustand in den Zivilstatus begreift Rousseau seiner Zivilisationskritik zum Trotz als „Veredlung des Menschen“. Durch den Gesellschaftsvertrag werde die Natur des Naturzustands in eine neue Natur transformiert und dabei vervollkommnet, was Rousseau als Voraussetzung für das Funktionieren der Gesellschaft einführt. Diese Transformation vom Naturzustand in die bürgerliche Gesellschaft ermögliche, dass die körperlichen Triebe durch Vernunft unter Kontrolle gebracht werden. Durch den Gesellschaftsvertrag handle der Mensch statt auf der Basis des „Instinkts“ auf der Grundlage der „Gerechtigkeit“, die den „Handlungen die Sittlichkeit […] [verleiht], die ihnen zuvor mangelte“ (Rousseau 2003, S. 22).

Auch Rousseaus Konzeption des politischen *Bürgers* weist einen verkörperten Subtext auf. Der Körper im Gesellschaftszustand ist nicht mehr der natürliche, setzt der Gesellschaftszustand ja den Ausgang aus dem Naturzustand voraus. Dennoch wird der Umgang mit dem Körper – der für Rousseau im Gesellschaftszustand eine quasi zweite Natur annimmt – zu einem wichtigen Parameter, um ein *politischer Bürger* zu werden. Nicht aber sollte der *Staatsbürger* seine Natur unterdrücken, sondern diese sollte sich durch Erziehung und Disziplinierung entfalten. Auch hier ist es das Verhältnis zwischen Subjekt und Körper, das zur Scheidelinie wird, um zu definieren, wer als Staatsbürger*in gilt und wer nicht, wenngleich es für Rousseau nicht die „Natur“ des Körpers, sondern der Umgang mit diesem ist, der die entscheidende Qualifikation zur An- oder Aberkennung der Staatsbürgerschaft bedeutet. Als politischer *undefined* *Bürger *gilt ihm nur der Mann, „der gute Sohn, der gute Gatte, der gute Vater“ (Rousseau 1922, S. 336), die Frau bleibt Untertanin und nimmt als Angehörige des Volkes nicht teil an der politischen Souveränität. Die Begründung dafür findet Rousseau in vermeintlich naturgegebenen Geschlechterdifferenzen. Anders als Männern gelinge es Frauen nicht, sich aus ihrem (Geschlechts‑)Körper herauszulösen, was für Rousseau gerade Bedingung dafür ist, ein politisches Subjekt zu werden. „Der Mann zeigt seine Mannheit nur in gewissen Augenblicken, die Frau dagegen bleibt Frau ihr ganzes Leben […] hindurch. Unaufhörlich wird sie an ihr Geschlecht gemahnt“ (Rousseau 1922, S. 331). Da die Frau durch ihre Konstitution abhängig sei, könne sie nicht wie der männliche Bürger aufgeklärt und erzogen werden, sondern müsse „dressiert“ werden (Appelt 1999, S. 69).

Wie Christine Klapeer argumentiert, nimmt in Rousseaus Bestimmung des *Staatsbürgers *Sexualität eine gewichtige Rolle ein (Klapeer 2014, S. 125–126). Für Rousseau ist die „Selbstbeherrschung“ (Rousseau 1922, S. 398) eine wichtige Voraussetzung, um ein *Bürger* zu werden; dieser müsse „Herr seiner Person“ sein (Rousseau 1922, S. 583). Gerade dies könne bei Frauen nicht sichergestellt werden, da Rousseau zufolge weibliche Sexualität eine potenziell unkontrollierbare Kraft sei. Diese müsse durch eine „natürliche Scham“ der Frauen gezügelt und in der heterosexuellen Ehe gebannt werden (Rousseau 1922, S. 327). Erst auf diese Weise verliere die weibliche Sexualität ihre Gefährlichkeit und könne dem öffentlichen Wohl dienlich werden, freilich nicht, indem auch Frauen zu „Herrinnen ihrer Person“ und als solche zu Staatsbürgerinnen werden, sondern indem sie die Rolle der Ehefrau und Mutter übernehmen. Die Bezwingung des Körpers erweist sich bei Rousseau als „Konstitutionsbedingung der Realisierung der politischen Freiheit des männlichen Staatsbürgers“ (Klapeer 2014, S. 137), während heteronormativ-patriarchale Imaginationen von sexualisierten Körpern den Ausschluss von Frauen aus dem Staatsbürgerstatus legitimieren (Appelt 1999, S. 69; Klapeer 2014, S. 132). Auch bei Rousseau fungieren männlich-heteronormative Imaginationen über weibliche Körper und Sexualität also als Begründung für politische Ausschlüsse, die aber gerade weil sie auf den vermeintlich natürlichen leiblichen Körper rekurrieren, zugleich entpolitisiert bleiben.

Mit Immanuel Kant setzt eine neue Begründung von Staatsbürgerschaft ein: An die Stelle naturrechtlicher Argumentationen tritt die moralisch-sittliche Vernunft. Gleich aber bleibt, dass sich auch Kant dabei vorgeblich präpolitischer Körperkonstruktionen bedient. Kant bestimmt den Menschen als Vernunftwesen, allerdings nicht aus der Perspektive einer „physiologischen“, sondern einer „pragmatischen“ Anthropologie. Diese stelle nicht die Frage, „was die *Natur* aus Menschen macht“ (Kant 2000, S. 1), sondern was der Mensch, „als frei handelndes Wesen, aus sich selbst macht, oder machen kann und soll“ (Kant 2000, S. 1). Konstitutiv für den Menschen sei, „daß er einen Charakter hat, den er sich selbst schafft; indem er vermögend ist, sich nach seinen von ihm selbst genommenen Zwecken zu perfektionieren; wodurch er, als mit *Vernunftfähigkeit* begabtes Tier (*animal rationabile*), aus sich selbst ein *vernünftiges* Tier (*animal rationale*) machen kann“ (Kant 2000, S. 257). Hier wiederum schreibt Kant der Natur eine ihr eigene teleologische Kraft zu: Die Natur „wolle, daß jedes Geschöpf seine Bestimmung erreiche; dadurch, dass alle Anlagen seiner Natur sich zweckmäßig für dasselbe entwickeln“ (Kant 2000, S. 267). Vor allem durch die Erziehung müsse der Mensch lernen, „die Naturanlagen proportionierlich zu entwickeln“ (Kant 1803, S. 13). Diese bedeute Zivilisierung, Moralisierung und Bezähmung der „Wildheit“ (Kant 1803, S. 9).

Aus einer körpertheoretischen Perspektive lässt sich zeigen, dass Kant seiner Prämisse der „pragmatischen Anthropologie“ nur inkonsequent Folge leistet. Denn in der Frage, wer als politisches Subjekt gilt, rekurriert er auf Unterschiede zwischen Menschen, die er als natur*gemacht* bestimmt. Seine Definition des politischen Subjekts, das sich durch Vernunft, Freiheit, Autonomie und Moralität auszeichnet, unterläuft er mit seiner „physiologischen Anthropologie“ und findet genau in dieser seine Begründung, warum nur weiße Männer aktive Staatsbürger sein könnten und alle anderen Menschen zu einem Leben in Inhärenz bestimmt seien.

Nur die Fähigkeit zur Stimmgebung macht die Qualifikation zum Staatsbürger aus; jene aber setzt die Selbständigkeit dessen im Volk voraus, der nicht bloß Teil des gemeinen Wesens, sondern auch Glied desselben, d.i. aus eigener Willkür in Gemeinschaft mit anderen handelnder Teil desselben sein will. […] [D]er Dienstbote […], der Unmündige […]; alles Frauenzimmer und überhaupt jedermann, der nicht nach eigenem Betriebe, sondern nach der Verfügung anderer […] genötigt ist, seine Existenz (Nahrung und Schutz) zu erhalten, entbehrt der bürgerlichen Persönlichkeit, und seine Existenz ist gleichsam nur Inhärenz. (Kant 1959, S. 137)

Obwohl nach Kant zufolge zwar alle Menschen, „die ein Volk ausmachen“, gleich sind, könne es dennoch qua Geschlecht und Klasse einen ungleichen Rechtsstatus zwischen ihnen geben, wie er dies in seiner Unterscheidung zwischen Aktiv- und Passivbürgern argumentierte: „Staatsbürger, nicht bloß Staatsgenosse zu sein, dazu qualifizieren sich nicht alle mit gleichem Recht“ (Kant 1959, S. 138). Aus dem passiven Bürgerstatus „folgt nicht das Recht, auch als *aktive* Glieder den Staat selbst zu behandeln, zu organisieren oder zur Einführung gewisser Gesetze mitzuwirken“ (Kant 1959, S. 138). Dass Frauen also auch passive, nicht aber aktive Staatsbürgerinnen sein können, steht für Kant nicht im Widerspruch zu dem allgemeinen Gleichheitspostulat, da er seine Theorie auf der für ihn selbstverständlichen Annahme von Geschlecht als natürlicher Ungleichheitsgröße aufbaut (s. a. Appelt 1999, S. 67). Auch bei Kant sind es daher Imaginationen über die leiblichen Körper und vermeintlicher geschlechtlicher Unterschiede ebendieser, die konstitutiv für seine Bestimmung des politischen Subjekts sind.

Nicht nur gründet Kant seine Definition des politischen Subjekts in Annahmen von natürlich unterschiedenen Geschlechtskörpern. Noch grundlegender beruht seine Konzeption der reinen Vernunft, die er zum Charakteristikum politischer Subjektivität erhob, auf einer impliziten Körperpolitik. Kant propagiert ein Subjektverständnis, das dieses in einen rationalen und einen physischen Anteil unterteilt, denn die reine Vernunft ist als vollständig entkörperte Vernunft konzipiert. Wie Jane Flax argumentiert, muss auch diese Konzeption des Subjekts als entkörpertes Vernunftwesen als Phantasma dechiffriert werden. Denn die „reine Vernunft“ lässt sich nicht von ihrer Eingebettetheit in den Körper trennen. „The boundaries between psyche and soma or between desire, embodiment, and thought are not impermeable or fixed. […] Unconscious processes operate outside and by different rules from those of rational thought. Their effects on rational ones can never be transparent or fully controlled“ (Flax 1993, S. 83). Auch in dieser Konzeption einer entkörperten Vernunft dienen Annahmen über naturgegebene verschiedene Geschlechtskörper als Ermöglichung der Aufrechterhaltung dieses Phantasmas. Die zeitgenössische Gleichsetzung von Frauen und Körpern ist die Kehrseite von Kants Konzeption einer entkörperten reinen Vernunft:

Identifying women with the body and the particular are two of the necessary conditions for the possibility of conceptualizing a disembodied and universal faculty (e.g. […] Kant’s pure reason). Women = the body; therefore men are not possessed/determined by it. The effects of male embodiment and social experiences on reason and its products are defined out of existence. With the contaminating effects of difference suppressed or denied, reason can take on its unitary and universal appearance. (Flax 1993, S. 85)

Neben der Vergeschlechtlichung von Körpern nimmt in Kants Konstruktion politischer Subjektivität die Rassifizierung von Körpern eine zentrale Rolle ein. Mit dieser begrenzt er den Kreis der aktiven *Bürger* auf weiße Männer. Kant lässt sich als „father of the modern concept of race“ interpretieren (Mills 1997, S. 70), denn er operiert explizit mit einem biologistischen „Rassebegriff“. Er geht von der Existenz vier verschiedener „Rassen“ aus (Kant 1968, S. 432), die er nach im Blut unterschiedlich vorhandener Säfte definiert (Kant 1968, S. 432). Als Unterscheidungsmerkmal benennt er physiologische Merkmale wie Haut‑, Haar- Augenfarbe, Schädelform, das „Naturell“ sowie den „Zivilisationsgrad“ (Kant 1968, S. 433). Die vermeintliche „Überlegenheit“ weißer Menschen leitet er aus deren angeblich vollkommener Organisation des Körpers ab. Vermeintliche körperliche Unterschiede, die Kant als naturgegeben und vorpolitisch konstruiert, sind die Voraussetzung dafür, dass er seine politik-theoretischen Abhandlungen zur Frage, was der Mensch als *freies Wesen* machen *kann*, nur auf weiße Männer beziehen kann. Schwarze Menschen assoziiert er mit Natur und spricht ihnen den Status freier, politischer Subjekte ab. Emmanuel Chukwudi Eze bringt den rassistischen Subtext von Kants Subjektverständnis wie folgt auf den Punkt: „The black person […] can accordingly be denied full humanity, since full and ‚true‘ humanity accrues only to the white European. For Kant European humanity is *the* humanity *par excellence*“ (Eze 1997b, S. 121). Diese Unterschiede in der Vernunft wie im Menschsein leitet Kant aus seinen Imaginationen über die Körper ab.

Kant’s position on the importance of skin color not only as encoding but as *proof* of this codification of rational superiority or inferiority is evident in a comment he made on the subject of the reasoning capacity of a „black“ person. When he evaluated a statement made by an African, Kant dismissed the statement with the comment: „this fellow was quite black from head to foot, a clear proof that what he said was stupid“. It cannot, therefore, be argued that skin color for Kant was merely a physical characteristic. It is, rather, evidence of an unchanging and unchangeable moral quality. „Race“, then, in Kant’s view, is based upon an ahistorical principle of reason (*Idee*) and moral law. (Eze 1997b, S. 119)

Die Rassifizierung von Körpern dient Kant zur Legitimation der Annahme, dass in der „biologischen Natur“ Unterschiede angelegt seien, die dazu führen würden, dass nur weiße Menschen sich aus dem Zustand der Unmündigkeit befreien und den Status des Menschlichen erreichen könnten. Schwarzen Menschen spricht Kant gerade wegen ihrer Körper die Fähigkeit der Autonomie und dementsprechend die Kompetenz, sich eine bürgerliche Verfassung zu geben, ab. Indem er Schwarzen Menschen den politischen Subjektstatus verweigert und weißen einen „Führungsanspruch in geistiger und militärischer Hinsicht“ zugesteht (Hentges 1999, S. 218), liefert Kant mit seiner politischen Theorie eine politik-theoretische Rechtfertigung kolonialer Unterwerfung und Ausbeutung (Mills 2005).

Obwohl Kant also das politische Subjekt als rein entkörpertes Vernunftwesen definiert, sind es in seiner Argumentation die Körper jener, die ihm als „Andere“ und „Nichtsubjekte“ gelten, die begründen sollen, warum diese nicht als politische Subjekte qualifiziert sein können. Diesen Widerspruch löst Kant, indem er die angeblichen Unterschiede der leiblichen Körper im (ebenso angeblich) vorpolitischen Reich der Natur ansiedelt.

Es lässt sich schlussfolgern, dass auch für die Legitimation des politischen Subjektstatus Körpern eine entscheidende Rolle zukommt. In der modernen westlichen Politischen Theorie werden die Subjekte der politischen Vernunft als „körperlose Denkmaschinen“ konzipiert (Benhabib 1992a, S. 12). Nur wem zugestanden wird, seinen Körper mittels Vernunft kontrollieren und beherrschen zu können, gilt als politisches Subjekt. Das freie politische Subjekt der westlichen Moderne ist immer auch ein *vom Körper befreites Subjekt*. „Political freedom is rooted in opposition to confinement by the body, by natural needs and desires, by the body politic“ (Brown 1988, S. 180), so beschreibt Wendy Brown daher die Rolle des Körpers in der Definition des modernen westlichen politischen Subjekts.

Dass der Körper als Antipode des politischen Subjekts konzipiert wird, macht dessen Selbst zu einem geteilten; es wird das Andere des materiellen Körpers:

The body is not the subject person—because that is the mind—but rather an object which belongs to that subject. The body is therefore alienated and fetishized. The body is not literally exterior to the person, in the manner of other objects of property […], and yet it is regarded as a form of external housing fort e immaterial mind. It is baser, it is mundane, it is inferior, and it is natural. And yet it is a necessary condition of the person. (Naffine 1998, S. 202)

Die der Konstruktion des modernen westlichen politischen Subjekts zugrunde liegende „‚Entkörperung des Menschen‘“ (Kerchner 1999, S. 61) erweist sich als Machttechnik, der zufolge weiße, heteronormative, nichtbehinderte Männer zur „körperlosen Norm“ werden konnten, und allen Anderen politische Handlungsfähigkeit gerade aufgrund einer vermeintlichen Minderwertigkeit oder Mangelhaftigkeit ihrer Körper abgesprochen werden konnte. Der weiße, männliche, heteronormative, nichtbehinderte Körper konnte in der Konstruktion des politischen Subjekts unsichtbar werden, indem er zur präpolitischen Norm wurde. „The reality is that one can pretend the body does not matter only because a particular body (the white male body) is being presupposed as the somatic norm. In a political dialogue between the owners of such bodies, the details of their flesh do not matter since they are judged to be equally rational, equally capable of perceiving natural law or their own self-interest“ (Mills 1997, S. 53). Die zugeschriebene Inferiorität weiblicher, Schwarzer, queerer, behinderter Körper wird zur Begründung dafür, dass als politische Subjekte nur jene galten, die vermeintlich körperlos waren. „Slaves, foreigners, women, the conquered, children, the working classes have all been excluded from political participation, at one time or another, by their bodily specifity“ (Gatens 1997, S. 83).

Der Körper entschwindet also nicht aus der Konstruktion des politischen Subjekts; er wird zu einem konstitutiven Element des politischen Subjektstatus – nicht aber in seiner Präsenz, sondern in seiner Abwesenheit: als kontrollierter, disziplinierter, bezwungener, beherrschter. Obgleich also das politische Subjekt in der westlichen Politischen Theorie als entkörpertes präsentiert wird, lassen sich Bedingungen des politischen Subjekts ausmachen, die *am Körper, im Körper, mit dem Körper und durch den Körper* erfüllt sein müssen, um als politisches Subjekt gelten zu können.

## Körper und Politik

Was als Gegenstand und Sinn von Politik definiert wird, wird in der Politischen Theorie seit jeher kontrovers diskutiert. Wie insbesondere feministische Theoretiker*innen (Lang 2004; Sauer 1997) deutlich gemacht haben, ist für die Bestimmung von Politik in der modernen westlichen Denktradition die Grenzziehung zwischen Öffentlichkeit und Privatheit grundlegend: Dass der Kanon der Politischen Theorie Politik mit Öffentlichkeit gleichsetzt und alles, was im Privaten angesiedelt wird, als „unpolitisch“ ausspart, wurde daher von feministischen politischen Theoretiker*innen als machtvoller Mechanismus der Einengung von Politik kritisiert. Im Folgenden werde ich darlegen, wie der Radius dessen, was als „Politik“ gilt, auch durch Ausschlüsse von Körpern eingehegt wird. Dies werde ich anhand von drei Theoriesträngen zeigen, die „Politik“ sehr unterschiedlich definieren: Hannah Arendt, Jürgen Habermas und John Rawls. Trotz aller Unterschiede in der Definition von Politik, verbindet die drei Theoriestränge eine argumentative Gemeinsamkeit: Sie alle erachten körperliche Bedürfnisse und Erfahrungen als irrelevant für Politik und, so werde ich zeigen, verengen auf diese Weise das Verständnis dessen, was als Politik gilt.

Emblematisch für eine Konzeption von Politik als Gegenpol zu allem Körperlichen ist Hannah Arendts politische Theorie. Konstitutiv für diese ist eine klare Grenzziehung zwischen dem Privaten und dem Öffentlichen. Politik ist für Arendt eindeutig im Öffentlichen angesiedelt und kann erst beginnen, wenn alle „menschlichen Bedürfnissen und Lebensnotwendigkeiten“ in der „Sphäre des Haushalts“ organisiert sind (Arendt 2002, S. 40). Politik umfasst also gerade nicht alltägliche Lebensnotwendigkeiten oder die materiell-körperliche Reproduktion; diese gelten Arendt als „präpolitisch“ (Arendt 2002, S. 41). Öffentlichkeit ist für Arendt die Sphäre der Politik, gerade weil die Menschen in dieser ihren körperlich-materiellen Bedürfnissen enthoben sind. Arendt exkludiert nicht nur alles Körperliche aus der Politik, sondern ordnet Politik und Körper auch in einer hierarchischen Weise an. Politisches Handeln bestimmt sie als höchste menschliche Tätigkeit, Arbeit, die „von der Notdurft des Körpers bestimmt und von seiner Mühsal erzeugt“ werde (Arendt 2002, S. 112), als „das unterste Niveau menschlichen Tätigseins“ (Arendt 2002, S. 150).

Als Sinn von Politik gilt Arendt Freiheit (Arendt 2003, S. 35). Diese konzipiert Arendt weder solipsistisch noch individualistisch, sondern intersubjektiv. Freiheit und Politik entstehen erst im Miteinanderhandeln und setzen Pluralität voraus (Arendt 2003, S. 39). Aufgrund der ihr zugrunde liegenden Pluralität der Menschen zeichnet sich Politik dadurch aus, dass weder ihre Richtung noch ihre Konsequenzen vorab bestimmbar sind. Erst im Miteinander nimmt Politik Gestalt an. Politisches Handeln ist Neuanfang und erfordert daher die Bereitschaft, sich auf ein Handeln mit Anderen einzulassen, ohne dass dieses a‑priori festgelegt werden kann.

Die Konzeption von Politik als dem Körper und dem Privaten Entgegengesetztes evozierte insbesondere bei feministischen und rassismuskritischen Theoretiker*innen Kritik (u. a. Benhabib 1992b; Honig 1995; Zerilli 1995). Als eine der Hauptkritiken wurde vorgebracht, dass der Ausschluss von Körperlichkeit und reproduktiver Arbeit aus der Politik keineswegs zu einer öffentlichen Sphäre der Freiheit und Pluralität führe, sondern vielmehr dazu diene, dass diese eine „patriarchalisch-elitär beschränkte Öffentlichkeit“ bleibe (Lettow 1996, S. 239). Arendts Konzeption von Politik wurde als beschränkt kritisiert, da es nicht nur all jene ausschließt, denen es nicht möglich ist, sich von materiellen und körperlichen Notwendigkeiten zu befreien, sondern auch, da es alle Bereiche und Erfahrungen, die mit Körpern verbunden sind, als unpolitisch definiert: die soziale Frage und die gesamtgesellschaftliche Arbeitsteilung, die Sorge um Körper und körperliche Bedürfnisse und Erfahrungen wie Schmerz oder Gewalt.

Nicht aber nur verengt Arendt durch den Ausschluss von Körpern ihre Konzeption von Politik. Darüber hinaus führt die Ansiedlung von Körpern im Präpolitischen dazu, dass Arendts eigener Anspruch eines Neudenkens von Politik auf halbem Wege stehen bleibt. Arendt denkt Politik nicht von autonomen Subjekten ausgehend, sondern als offenen Prozess der Partizipation, in der die eigenen Perspektiven sich im Miteinander mit Anderen transformieren (Arendt 2003, S. 52). Politik umfasst daher die Bereitschaft, sich auf andere Menschen und Perspektive einzulassen, um gemeinsam neue Perspektiven entwickeln zu können. Auf diese Weise entwickelt Arendt ein post-souveränes Konzeption von Politik, mit dem sie sich auch von liberalen politischen Theorien abgrenzt (Meyer 2013). Politik ist, da sie an die Pluralität und Anwesenheit der Anderen gebunden ist, post-souverän: Sie braucht die Anderen und transformiert das Ich durch die Anderen. Politik enthält notwendig Unvorhersehbares und erfordert daher auch Mut, sich auf das „Wagnis“ des Neuen einzulassen (Meyer 2013, S. 45).

Trotz Arendts Anspruch, die liberale Vorstellung eines autonomen, solipsistischen Individuums zu überwinden, behält sie in Bezug auf Körper jedoch genau deren Perspektive bei: Denn Körper gelten Arendt als „eigenes“ und „abgeschlossenes“ und als solches verbannt sie sie aus der Politik. Wie Judith Butler in jüngeren Arbeiten (2018) gezeigt hat, hätte Arendt aber ihren Anspruch einer postsouveränen Theorie der Politik noch radikalisieren können, wenn sie Körper als ebenso plurale und mit anderen verwobene Gefüge mit in die Konzeption von Politik aufgenommen hätte: Gerade wenn Körper nicht als präpolitisch gesetzt werden, sondern Subjekte als notwendig verkörperte Wesen und Körper als stets mit anderen Körpern verwobene in eine Theorie der Politik integriert werden, lässt sich Arendts Anspruch eines postsouveränen Politikverständnisses erweitern. Denn das politische Handeln in der Öffentlichkeit als Handeln zwischen Menschen ist immer auch ein Handeln zwischen Körpern (Butler 2018, S. 64). Die Performativität des politischen Handelns, die Arendt herausstreicht, „umfasst nicht nur die Sprache, sondern auch die Ansprüche körperlicher Handlungen, Gesten, Bewegungen, der Versammlung, der Persistenz und des potenziell Gefährdetseins durch Gewalt“ (Butler 2018, S. 102). Politik, die aus dem Miteinander hervorgeht, ist immer auch ein Miteinander der Körper (Butler 2018, S. 104). Konsequenterweise entsteht das Neue und Unvorhersehbare, das Politik nach Arendt ausmacht, nicht nur durch das Zusammenkommen von entkörperten Subjekten, sondern auch von sich in der Öffentlichkeit versammelnden Körpern. Das Unvorhersehbare der Politik kann, wie Butler über Arendt hinausgehend argumentiert, gerade durch Körper begründet werden, wenn Körper nicht als präpolitisch der Politik vorgelagert werden, sondern als Teil von Politik anerkannt werden. Das Miteinandererscheinen im öffentlichen Raum ist auch ein körperliches Erscheinen, das gerade weil es verkörpert ist, Momente enthält, die dem Ich entzogen bleiben: „Was das politische Handeln angeht, muss ich also den anderen in einer Weise erscheinen, die ich nicht kennen kann, und folglich wird mein Körper von Perspektiven bestimmt, die ich nicht einnehmen kann, die aber gewissermaßen mich einnehmen“ (Butler 2018, S. 104). Diese Perspektiven der Anderen können für das Ich nie vollständig transparent werden. Folglich bedeutet Politik auch das Sich-Einlassen in und Aussetzen an körperliche Imaginationen, die die Anderen im Miteinanderhandeln an das Ich richten. Eine Perspektive, die Körper – nicht wie Arendt selbst – als Gegenpol von Politik konzipiert, könnte folglich den Radius von Politik und Arendts eigenen Anspruch eines postsouveränen Politikverständnisses erweitern.

Freilich unter ganz anderen Vorzeichen wie bei Arendt enthält auch das Verständnis von Politik, das Jürgen Habermas in seiner deliberativen Demokratietheorie entwickelt, Einschränkung, die ebenso als Konsequenz des Ausschlusses von Körpern aus dem Feld der Politik dechiffriert werden können. Ähnlich wie für Arendt ist auch für Habermas die Öffentlichkeit zentraler Ort von Politik (Habermas 1962, S. 323). Das „Prinzip der Öffentlichkeit“ gilt Habermas als Garant dafür, dass „jede andere Gewalt als die des besseren Arguments“ ausgeschlossen wird (Habermas 1969, S. 123). Als Modus der Politik gilt Habermas die Vernunft. Diese solle in deliberativen Prozessen garantieren, dass sich der „eigentümliche zwanglose […] Zwang des besseren Arguments“ (Habermas 1973, S. 240) durchsetzt und auch in pluralen Gesellschaften vernünftige Einigung garantiert werden kann. Die in der Lebenswelt verortete „ideale Sprechsituation“, in der kommunikative Vernunft handlungsleitend ist, wird für Habermas zum Grundstein seines vernunftzentrierten Politikverständnisses: Aus der Prämisse, wonach Verständigung „als Telos der menschlichen Sprache inne[wohnt]“ (Habermas 1981, S. 387), leitet er ab, dass Politik auf kommunikativer Vernunft beruhen müsse, wenn sie demokratisch sein will.

Durch die Gleichsetzung von Politik und Öffentlichkeit muss auch bei Habermas all das, was als unvernünftig, partikular und verkörpert gilt, in das vorpolitische Private verschoben werden (Allen 2008, S. 104; Young 1990, S. 97). Habermas’ Konzeption von Politik erweist sich daher ebenso als begrenzt, da seine Annahme, dass es ein allgemein geteiltes Interesse gebe, das im Prozess der Deliberation mittels Vernunft freigelegt werden könne, alle „privaten“ Belange ausschließt, die mit der Versorgung und Pflege von Körpern verbunden sind. Diese Tätigkeiten können nicht qua Vernunft organisiert werden und in ihnen lässt sich auch kein allgemeines, geteiltes Interesse finden – nicht zuletzt da sie in Gegenwartsgesellschaften durch vergeschlechtlichte, heteronormative, ability-zentrierte, rassistische Ungleichheitsverhältnisse organisiert werden, wie sich etwa in der globalen, vergeschlechtlichten Arbeitsteilung zeigt. Die gesellschaftliche Organisation der Versorgung, Pflege, Unterstützung und Begleitung von Körpern muss aus Habermas’ Konzeption von Politik ausgeschlossen bleiben (Fraser 1985). Körperpolitiken, die festlegen, wer sich um alte und kranke Körper kümmert, wer als „normaler“ Körper gilt und wie Rechte an Körpernormalisierungen gebunden sind, entschwinden in der Habermas’schen Gleichsetzung von Politik und entkörperter Vernunft ins Private.

Darüber hinaus erweist sich Habermas’ Verständnis der „allgemeinen“ Vernunft als „elitist and exclusive“ (Young 1997a, S. 63), wie Iris Marion Young kritisiert. Das, was als „allgemeine Vernunft“ gilt, ist keineswegs derart universell, wie Habermas dies annimmt, sondern vielmehr eine Sedimentierung von weißen, maskulinen, heteronormativen, ability-zentrierten Lebensweisen (Young 1997a, S. 66). Auch diese Verengung des Politikverständnisses, das auf der Annahme einer „allgemeinen Vernunft“ beruht, resultiert aus der Entkörperung von Politik. Denn mit dem Postulat, dass der Modus von Politik eine „allgemeine Vernunft“ sei, schreibt Habermas die euro- wie androzentrische Fiktion eines neutralen, objektiven, entkörperten Standpunkts als Grundlage für politisches Handeln fort. Habermas konzipiert die Teilnehmenden des politischen Diskurses als reine Vernunftwesen, die jeder Körperlichkeit enthoben sind. Damit beruht Habermas’ Theorie jedoch auf der Illusion, dass es überhaupt möglich sei, einen derartigen „allgemeinen“, „entkörperten“ Standpunkt einzunehmen. Mit Ramón Grosfoguel lässt sich an dieser Stelle gegen Habermas’ Verständnis von Vernunft einbringen, dass dieses auf einer *unmöglichen* Abstraktion von „‚body-politics of knowledge‘“ beruht (Grosfoguel 2007, S. 213):

Nobody escapes the class, sexual, gender, spiritual, lingustic, geographical, and racial hierarchies of the „modern/colonial capitalist/patriarchal world-system“. By delinking ethnic/racial/gender/sexual epistemic location from the subject that seaks, western philosophy and sciences are able to produce a myth about a truthful universal knowledge that covers up, that is, conceals who is speaking as well as the geo-political and body-political epistemic location in the structures of colonial power/knolwedge from which the subject speaks. (Grosfoguel 2007, S. 213)

Eine derartige Konzeption von Politik auf der Basis des Primats der Vernunft führt dazu, dass die Modi von Politik verengt werden und körperliche, affektive, „nichtrationale“ Aspekte des Selbst präpolitisch bleiben müssen, wie auch Amy Allen kritisiert: „[T]he problem is not that Habermas stresses the rational potential implicit in processes of argumentation, it is that he overemphasizes this potential while simultaneously underemphasizing the other – nonrational, bodily, affective, concrete – aspects of our selves“ (Allen 2008, S. 153). Ähnlich problematisiert Young, dass Formen des politischen Handels, die als leidenschaftslos und entkörpert – respektive als „vernünftig“ – gelten, privilegiert werden, während Ausdrucksformen, die als verkörpert und „unvernünftig“ gelten (wie etwa Emotionen), als partikular abgewertet werden (Young 1997b, S. 123).

Obwohl wiederum auf gänzlich anderen epistemologischen und theoretischen Prämissen beruhend, lässt sich auch für John Rawls’ Theorie konstatieren, dass diese ebenso durch die Ausblendung von Körpern den Radius dessen, was als politisch gilt, verengt. Rawls’ Grundfrage ist, wie „eine gerechte und stabile Gesellschaft von freien und gleichen Bürgern dauerhaft bestehen [kann], wenn diese durch ihre vernünftigen religiösen, philosophischen und moralischen Lehren voneinander getrennt sind“ (Rawls 1998, S. 35). Als Antwort entwickelt er Grundsätze der Gerechtigkeit, die dem Kriterium standhalten sollen, dass sie „freie und vernünftige Menschen in ihrem eigenen Interesse in einer anfänglichen Situation der Gleichheit […] annehmen würden“ (Rawls 1975, S. 28). Bei Rawls ist es ebenso die Vernunft, die zum Grundmodus von politischem Handeln wird. Die Motivation der Kooperation der rationalen Akteur*innen (1975, S. 28) leitet er aus dem Interesse der Einzelnen ab, ihre je eigenen Vorteile sicherzustellen (Rawls 1975, S. 28.).

In einem fiktiven Urzustand siedelt Rawls sein berühmtes Gedankenexperiment an, mit dem er Verfahrensgerechtigkeit begründen möchte. In diesem sind allen Mitgliedern mittels eines „Schleier[s] des Nichtwissens“ (Rawls 1975, S. 159) jegliches Wissen über ihre Positioniertheit vorenthalten. Niemand kennt „seinen Platz in der Gesellschaft, seine Klasse oder seinen Status; ebenso wenig seine natürlichen Gaben, seine Intelligenz, seine Körperkraft usw.“ (Rawls 1975, S. 160). Leitend soll ausschließlich der Standpunkt des verallgemeinerten Anderen sein. Die Abstraktion von allen konkreten Erfahrungen und Identitäten solle verhindern, dass die beteiligten Parteien ihre Entscheidung nur an ihrem eigenen Nutzen und Vorteil ausrichten. Die auf diese Weise hervorgebrachte Konzeption von Gerechtigkeit würde gewährleisten, dass das Ergebnis „nicht von willkürlichen Zufälligkeiten oder gesellschaftlichen Kräfteverhältnissen beeinflusst“ werde (Rawls 1975, S. 142).

Da Rawls’ Konzeption von Gerechtigkeit die Abstraktion von konkreten Positionierungen voraussetzt, ist es konsequent, dass er auch Körpern keinerlei Bedeutung für eine Politik der Gerechtigkeit zumisst. Dies hat allerdings zur Konsequenz, dass es ihm nicht gelingen kann, eine umfassende Konzeption von Gerechtigkeit vorzulegen, wie ability-zentrismuskritische, rassismuskritische, feministische und queere Theoretiker*innen gezeigt haben (u. a. Arneil 2009; Benhabib 1992a; Nussbaum 2010). Indem Rawls seine Theorie auf der Abstraktion von konkreten Erfahrungen aufbaut, nimmt er an, dass gesellschaftliche Ungleichheiten sich in einem Gedankenexperiment ablegen ließen. Damit negiert er, dass Ungleichheitserfahrungen sich in einer Weise in Körpern einschreiben, von denen nicht vollständig abstrahiert werden kann. Seyla Benhabib diagnostiziert Rawls’ Theorie daher eine „‚epistemische Inkohärenz‘“, da diese auf einer Konzeption von politischen Subjekten beruhe, die „als ‚losgelöst‘, ‚körperlos‘ und mit der Fähigkeit begabt dargestellt werden, vom Standpunkt eines jeden anderen hinter einem ‚Schleier des Nichtwissens‘ zu argumentieren“ (Benhabib 1992c, S. 185).

Dass die Annahme, Menschen könnten im politischen Handeln ihre Körper „ausblenden“, zu einer Verengung von Gerechtigkeit beiträgt, weil so Formen der Ungerechtigkeit, die sich in Körper einschreiben, entpolitisiert bleiben müssen, hat Shatema Threadcraft aus einer rassismustheoretischen Perspektive in „*Intimate Justice*“ (2016) verdeutlicht. Threadcraft zeigt auf, dass in einer Gerechtigkeitskonzeption, wie Rawls sie vorschlägt, ganz grundlegende *politische verkörperte Erfahrungen* ausgespart werden müssen. Dies macht Threadcraft deutlich, indem sie argumentiert, dass Unrecht, das aus Rassismus und Sklaverei folgt, sich nicht nur auf ökonomischer und rechtlicher, sondern auch auf einer körperlichen Ebene manifestiert. Sklaverei ist auch ein System der Ausbeutung Schwarzer Körper und der Enteignung körperlicher Reproduktion, Sicherheit, Gesundheit und Integrität von Schwarzen Menschen, um die Privilegien von weißen Körpern zu sichern, deren Auswirkungen bis in die Gegenwart sich in Körper einschreibt.

Whites have not only been advantaged with regard to positions of social advantage; other important aspects of white advantage concern aspects of the body and human life, specifically in their efforts to reproduce, to keep the body alive, healthy, and emotionally well adjusted. Racial domination has also allowed whites to hoard the benefits of the material labor necessary to maintain life and to secure the physical and emotional needs of the body itself, as, tragically, the benefits of the forced and coerced labor of black women were among the advantages that accrued to the white body as a consequence of racial domination. Racial domination has also advantaged whites with regard to bodily integrity and reproductive health, that is, it has allowed whites to hoard the benefits not only of the labors that typically fall to women, but also of state-sponsored, positive liberty-style support and protection for women’s own unique bodily needs. (Threadcraft 2016, S. 153)

Wie diese verkörperte Ungerechtigkeit kompensiert werden kann, lässt sich mit Rawls’ Gerechtigkeitskonzeption nicht beantworten. Ja, vielmehr noch, kann aufgrund der Aussparung von Körpern, wie sie Rawls’ Theorie zugrunde liegt, diese Frage gar nicht gestellt werden. Indem Rawls’ Theorie diese auch verkörperten Dimensionen von Ungerechtigkeit nicht nur ausblendet, sondern gar nicht zu theoretisieren in der Lage ist, bleibt sie daher nicht nur begrenzt, sondern schreibt genau jene *politischen* Formen der Ungerechtigkeit fort, die auch durch die Körper hindurchgehen und diese konstituieren.

In ganz unterschiedlichen Denktraditionen – der republikanischen, deliberativen und liberalen – lassen sich also Begrenzungen des Politikverständnisses nachweisen, die aus der Setzung von Körpern als „präpolitisch“ resultieren. Die Naturalisierung von Körpern fungiert folglich als Begrenzung dessen, was als politisch relevant gilt und worüber in der Politik verhandelt werden soll. Indem bestimmte Körper als Norm(alität) gesetzt werden und andere als Abweichung, indem Politik als entkörpert sowie als Gegenpol zum Körper konzipiert wird, indem Politik auf Vernunft reduziert wird, wird der Radius von Politik begrenzt.

## Abwesende Anwesende: Körper im modernen westlichen politischen Denken

Die vorangegangene körpertheoretische Archäologie der modernen westlichen Politischen Theorie konnte zeigen, dass Körper keineswegs abwesend sind; vielmehr wird in der Politischen Theorie auf Körper in vielfältiger Weise Bezug genommen: Als leibliche Körper, die eine politische Ordnung, den Ausschluss von Menschen aus dem Subjektstatus oder eine verengte Konzeption von Politik legitimieren sollen ebenso wie als politische Körper, die die leiblichen Körper in bestimmter Weise anordnen. Trotz dieser Bedeutung, die Körper in der modernen politischen Denktradition einnehmen, werden sie aber zugleich als „präpolitisch“ und „naturgegeben“ gesetzt – und gerade aus dieser Naturalisierung und Entpolitisierung erhalten sie ihre politische Bedeutung. Eine kritische körpertheoretische Perspektive in der Politischen Theorie einzunehmen, bedeutet folglich nicht, Körper in ein entkörpertes Terrain zu tragen, sondern Körperlichkeiten als *präpolitische* Setzungen zu dechiffrieren, die wiederum *politische* Anordnungen legitimieren.

Queere, feministische, ability-zentrismuskritische, rassismuskritische und postkoloniale Perspektiven können nicht nur dazu beitragen, die Bedeutung von Körpern im modernen politischen Denken sichtbar zu machen. Darüber hinaus findet sich hier auch ein umfassendes Archiv an Wissen, wie gerade durch die Einbeziehung von Körpern das Verständnis von politischer Ordnung, politischen Subjekten und Politik in emanzipatorischer Weise ausgeweitet werden kann, auf das hier nur noch ausblickend verwiesen werden kann: So hat Frantz Fanon die Dekolonisierung von Politik mit einer Dekolonisierung der Körper(-politiken) verbunden, um den gewaltförmigen Modus der eurozentrischen „Politik der Vernunft“ zu überwinden (Fanon 2013, S. 197, 2014, S. 267); aus feministisch-psychoanalytischer Sicht und ausgehend vom Körper wiederum hat Luce Irigaray (2001) eine Neudefinition von Demokratie vorgeschlagen, die jene Pluralität ins Zentrum rückt, die durch die von ihr als phallogozentrisch ausgewiesene liberale Demokratie verunmöglicht wird; Chris Beasley und Carol Bacchi haben über die Metapher des „*social flesh*“ eine politische Ethik der „embodied intersubjectivity“ entwickelt (2007, S. 290); Iris Marion Young (1997a) hat eine Konzeption demokratischer Öffentlichkeit entwickelt, die die Dichotomie von Vernunft und Körper hinter sich lässt; Martha Nussbaum hat eine Gerechtigkeitskonzeption bereitgestellt, welche die liberale entkörperte „Politik der Vernunft“ überwindet und auch körperliche Gesundheit und körperliche Integrität umfasst (Nussbaum 2010); Shatema Threadcraft (2016) hat eine Konzeption von reparativer Gerechtigkeit ausgearbeitet, die den bis in die Gegenwart anhaltenden gewaltförmigen Einschreibungen von Sklaverei und Kolonialismus in Schwarze Körper auch Rechnung trägt (Threadcraft 2016, S. 158–166); Judith Butler (2005, 2018) hat ausgehend von der Sozioontologie von Körpern ein Verständnis des Politischen als notwendig postsouverän und in und durch Körper performativ hervorgebracht konzipiert; und Gloria Anzaldúa (1987) hat ausgehend von Körpern als Erfahrungsorte der Fragmentierung, Transgression, Hybridität und Interkonnektivität einen neuen Sinnhorizont des Politischen entwickelt.

Wenn die Politische Theorie zukünftig die Auseinandersetzung mit Körpern von ihren Rändern in ihr Zentrum vordringen lässt, dann verspricht dies also einen zweifachen Erkenntnisgewinn: Auf einer analytischen Ebene führt eine derartige Politische Theorie zu Präzisierungen über Ausschlüsse und deren Legitimationsformen. Auf einer normativen Ebene birgt eine emanzipatorische Bezugnahme auf Körper das Potenzial eines Neudenkens von politischer Ordnung, politischen Subjekten und Politik, das bestehende Ausschlüsse und Begrenzungen zu überwinden versucht. Nicht nur angesichts der aktuellen Covid-19-Pandemie, die ganz unmittelbar die Verstrickungen von Politik und Körpern freilegt, sondern auch im Lichte der multiplen Krisen, auf die die Coronakrise auftrifft, erscheint mir dies dringlicher denn je. Denn auch die Krise des (neoliberalen) Kapitalismus, die Care-Krise, die Krise der Demokratie, das Erstarken von Rechtspopulismus und Rassismus sowie die sozioökologische Krise haben einen körperlichen Subtext: Sie haben unmittelbare Auswirkungen auf Körper, bedienen sich Imaginationen über politische wie leibliche Körper und setzen Körperpolitiken als Krisenbewältigungsmodus ein. Zugleich kämpfen politische Aktivist*innen bei Fridays for Future, in queer-feministischen Sorgestreiks oder in der Black-Lives-Matter-Bewegung auch ganz direkt für andere, für emanzipatorische Körperpolitiken.

Wie Körper und politische (An‑)Ordnungen in der Zukunft verknüpft werden, wird nicht in der Politischen Theorie entschieden, sondern ist Gegenstand gesellschaftlicher Aushandlungen und Ergebnisse gesellschaftlicher Kräfteverhältnisse. Politische Theorie kann jedoch diese gesellschaftlichen Prozesse begleiten und reflektieren. Wenn die Politische Theorie dies in einer Form unternehmen möchte, die demokratisch und emanzipatorisch ist, wird sie nicht umhinkommen, mit ihrer Tradition, Körper als präpolitisch zu verhandeln, einen Bruch zu vollziehen.

## Footnotes

1 Ich danke den Teilnehmer*innen des Bremer Kolloquium für Politische Theorie sowie Brigitte Bargetz für produktive Fragen und wichtige Anregungen zu einer früheren Fassung des Textes. Ebenso danke ich den anonymen Gutachter*innen für ihre Anmerkungen.

2 In dem vorliegenden Text wird an manchen Stellen bewusst die maskuline Form eingesetzt, um den geschlechtlichen Subtext der Figur ‚des Bürgers‘ zu verdeutlichen.

3 Dass Locke Anteilseigner der *Royal African Company* war, die 1672 das verbriefte Recht eines Monopols für den englischen Sklav*innenhandel erhielt, zeigt eine der direkten praktisch-politischen Konsequenz seiner politischen Theorie (McCarthy 2001, S. 628). Eine weitere wird in seiner Affirmation des Kolonialismus sichtbar: Diese leitete Locke aus seiner Annahme ab, dass „Amerika“ vor der Kolonialisierung ein „leeres“ von nomadischen „Wilden“ bewohntes Land gewesen sei, denen er absprach, Eigentum über dieses haben zu können (McCarthy 2001, S. 628).
